# Evaluation of an Invisible Illness Communication Strategy Curriculum Among Internal Medicine Interns

**DOI:** 10.7759/cureus.111890

**Published:** 2026-07-01

**Authors:** Maria Isabel Trejo Zambrano, Jillian Kyle, Rachel H Vanderberg

**Affiliations:** 1 Medicine, University of Pittsburgh Medical Center, Pittsburgh, USA; 2 Medicine, University of Pittsburgh School of Medicine, Pittsburgh, USA

**Keywords:** chronic pain, communication skills training, invisible illnesses, medical education, patient-provider communication

## Abstract

Introduction: Patient-physician communication in invisible illness is challenging. Invisible illnesses (e.g., fibromyalgia, myalgic encephalomyelitis/chronic fatigue syndrome, and disorders of gut-brain interaction) are clinical syndromes that are not readily visible or measurable by physical exam or diagnostic testing, and patients outwardly appear healthy. Patients with invisible illness often feel dismissed, while physicians often feel helpless or powerless when facing invisible illness. Invisible illness communication strategies can improve patient-physician relationships, patient and physician satisfaction, and patient outcomes, but are infrequently taught in medical education. We aimed to evaluate a novel invisible illness communication strategy curriculum using a pre-post study design.

Materials and methods: Internal medicine (IM) interns (n = 35) participated in monthly small-group sessions. The curriculum was an interactive, one-hour, case-based workshop introducing an original communication framework, i.e., the 5 Es (Evaluate, Empathize and validate, Educate, set Expectations, and Engage in care). Interns completed paired pre- and post-surveys assessing attitudes, comfort with communication skills using a five-point Likert scale, and perceptions of the curriculum. The data were analyzed with paired t-tests and Cohen’s dᶻ.

Results: All interns (35/35, 100%) completed both surveys. There was statistically significant improvement in comfort across all communication skills, with the largest effect size seen in increased comfort setting expectations (2.54 vs. 4.09, p < 0.01, Cohen’s dᶻ = 1.89). The curriculum was rated as excellent by 71% of interns. Helpful aspects of the curriculum included the practical framework, real-world phrasing, case-based discussion, and normalization of this challenging topic. Areas for improvement included dedicated time for practice conversations, additional invisible illness didactics, and emphasizing the patients’ perspective.

Conclusion: The invisible illness communication strategy curriculum was well-received by IM interns and increased comfort levels across communication skills.

## Introduction

Invisible illnesses (e.g., fibromyalgia, disorders of gut-brain interaction, and myalgic encephalomyelitis/chronic fatigue syndrome) are clinical syndromes that are not readily visible or measurable by physical exam or diagnostic testing, and the patient outwardly appears healthy [1]. Invisible illnesses are common; however, both patients and physicians, including trainees, report dissatisfaction with clinical encounters [2-8]. Patients often feel dismissed, while physicians often feel helpless [2-8]. Communication strategies are helpful in improving patient-physician relationships, patient and physician satisfaction, and patient outcomes, including pain, function, and quality of life [9-12]. Despite these benefits, communication strategies when working with patients with invisible illnesses are infrequently taught across all levels of medical education [8,13-18].

To our knowledge, invisible illness communication strategies have not been explicitly studied as a unified construct; however, related work exists within the literature addressing specific invisible illnesses, functional syndromes, noncancer pain, medically unexplained symptoms, and persistent physical symptoms. A recent study found that only 17% of residents felt confident or very confident in assessing chronic, noncancer pain, and 36% of residents rated their preparation for treating chronic, noncancer pain during residency as fair or poor [15]. Moreover, only 52% of residents reported that they frequently or very frequently believe reports of pain from patients with fibromyalgia [15]. A qualitative study found medical students and residents frequently described managing chronic pain as challenging and unrewarding, felt unsure about how to provide care, and identified a hidden curriculum suggesting patients with chronic pain have little educational value [4]. Chronic nociplastic pain, or central pain sensitization, is common in many invisible illnesses; however, invisible illness does not necessarily involve pain and may instead present with other debilitating symptoms such as fatigue, autonomic dysfunction, sleep difficulty, mood disturbances, or cognitive impairment (“brain fog”). A qualitative study exploring junior doctors’ knowledge and experience with medically unexplained symptoms found that junior doctors reported a substantial gap in training, negative attitudes of supervising doctors, and high levels of uncertainty, anxiety, and frustration regarding medically unexplained symptoms [17]. Similarly, another qualitative study exploring medical students’ perceptions of myalgic encephalomyelitis/chronic fatigue syndrome found no formal training, low levels of knowledge, and negative attitudes of supervising doctors [18].

Although invisible illnesses represent disparate medical conditions, patients report common patient-physician relationship issues across invisible illnesses. A systematic meta-synthesis examining 151 qualitative reports, including patients with Ehlers-Danlos syndrome, endometriosis, fibromyalgia, Gulf War syndrome, irritable bowel syndrome, long COVID, multiple chemical sensitivity, myalgic encephalomyelitis/chronic fatigue syndrome, postural orthostatic tachycardia syndrome, systemic lupus erythematosus, and vulvodynia, highlights the frequent invalidation, dismissal, or minimization of patient concerns and the subsequent negative consequences [19]. A qualitative study of 17 patient focus groups that included patients with over 10 types of chronic pain found that participants frequently reported negative experiences with their provider [5]. Issues included provider mistrust regarding patients’ reports of pain, providers accusing the patient of drug-seeking or of overexaggerating their symptoms, and providers not spending enough time with the patient. Participants also identified feeling undeserving of care and that they received lower quality care [5]. A narrative review examining patient perspectives in patients with encephalomyelitis/chronic fatigue syndrome found many of the same themes: frequent dissatisfaction with medical encounters, low-quality medical care, skepticism or minimization of complaints, physician reluctance to perform diagnostic testing, attribution of symptoms to mental health disorders, and lack of support [20]. Given the common challenges invisible illnesses present, including the subjective nature of complaints and subsequent patient-physician mutual mistrust, difficult-to-treat symptoms, prior negative patient experiences, and psychological stigma and shame, we believe invisible illness communication strategies can be approached as a unified construct [5,19-25].

Negative physician attitudes, lack of knowledge, and inadequate communication skills perpetuate pain invalidation, strain the patient-physician relationship, and are associated with negative patient outcomes, including increased pain, threats to moral integrity, impaired psychological and physical functioning, and reduced quality of life [26,27]. Furthermore, the combination of inadequate skills and the challenging nature of chronic pain can contribute to physician burnout [28]. Medical educators and professional organizations highlight the importance of bolstering chronic pain and medically unexplained symptom education [8,13,16,17,29,30], including communication skills [4,13,16,29]. Despite these calls to action, systematic reviews examining medical education curricula found that pain medicine and medically unexplained symptom curricula were inadequate [8,14,29,30]. Additionally, within these curricula, the most common topics were physiology, anatomy, and pharmacology, with little attention to communication skills [8,14,29,30].

Study objectives

As an initial step toward addressing this education gap, we developed a one-hour invisible illness communication strategy curriculum featuring a novel framework (the 5 Es). The primary objective of the curriculum was to increase participants’ comfort using a communication framework when working with patients with an invisible illness that facilitates a positive patient-physician relationship. A secondary objective was to increase participants’ confidence and sense of professional fulfillment when working with patients with invisible illnesses. We aimed to evaluate the invisible illness communication strategy curriculum with a pre-post survey design. Specific elements we examined included attitudes regarding invisible illness, acceptability of the curriculum, impact on comfort levels across various communication skills, specific helpful elements of the curriculum, and areas for improvement.

## Materials and methods

Setting and participants

The invisible illness communication strategy curriculum was delivered monthly during the 2024-2025 academic year by a single faculty member (RV) to small groups of approximately three to six internal medicine (IM) interns from a single academic institution during a one-hour ambulatory didactic session. No other faculty members delivered the curriculum. A total of 53 IM interns were eligible; however, only 35 IM interns participated in the curriculum. The 18 IM interns who did not participate in the curriculum had excused absences such as vacation and illness. We aimed to create a safe, open learning environment by normalizing the challenging nature of invisible illness and sharing personal experiences. The learning environment was also low stakes, as there were no formal or informal assessments tied to this curriculum.

Program description

The curriculum draws on elements of constructivism and reflective pedagogy [31]. The curriculum begins with a brief overview of invisible illness and a case-based discussion regarding a patient with a disorder of gut-brain interaction, which highlights common patient and provider challenges in invisible illness care. Next, the curriculum reviews the literature regarding the negative effects of pain invalidation and the positive effects of empathetic, patient-centered communication [10-12,19-30]. The 5 Es communication framework (Evaluate, Empathize and validate, Educate, set Expectations, and Engage in care) is introduced in the context of the patient journey. Evaluate & empathize and validate are emphasized during the diagnostic phase; educate and set expectations during diagnosis delivery and discussion; and engage in care during ongoing management. The framework highlights active listening, validation, patient education, focusing on functional outcomes, setting realistic goals, and using shared decision-making when creating a treatment plan. The curriculum explores how to discuss normal test results while still validating the seriousness and severity of symptoms. Additionally, the curriculum highlights how to discuss the importance of addressing mental health as part of the treatment plan while not attributing symptoms to mental health disorders. Appendix 1 presents a facilitator guide that includes key concepts, sample phrasing for each communication skill, and suggested responses to challenging patient statements that were also provided to participants as a take-home handout. Interns then practice applying the framework to the case, followed by questions and/or group discussion.

The 5 Es framework is an original, evidence-based framework developed from a review of communication literature, expert opinion, and patient feedback [2,9,12,28,32-37]. The 5 Es framework was initially developed by one author, RV, based on a review of the literature and patient feedback regarding a novel fibromyalgia clinic [37]. The framework was further refined through discussion with an expert in fibromyalgia, JK, and discussion and presentation to peers at a national conference who see patients with various invisible illnesses.

Program evaluation

All interns who participated in the curriculum were invited to complete anonymous, paired pre- and post-surveys. There were no incentives for participation in the surveys. Pre- and post-surveys were developed de novo for this curriculum (Appendix 2). Surveys were piloted with two additional faculty members with expertise in invisible illness and survey development to assess clarity, content, and face validity; however, there was no further psychometric validation. Surveys were completed via paper and pencil. Surveys were anonymous, and participants were asked to pair their surveys by stapling them together. Survey responses were transcribed into Microsoft Excel (Microsoft Corporation, Redmond, WA). Survey questions included demographics, attitudes regarding invisible illness, and comfort levels with various communication skills rated on a Likert scale (1 = strongly disagree, 2 = disagree, 3 = neutral, 4 = agree, 5 = strongly agree). Interns also rated the overall quality of the curriculum (1 = very poor, 2 = poor, 3 = average, 4 = good, 5 = excellent). Free-response items inquired about helpful aspects of the curriculum and areas for improvement. Descriptive statistics were generated for all variables. As this was a small, hypothesis‑generating study, all analyses were considered exploratory. Likert‑scale responses were treated as approximately continuous, as distributions were not markedly skewed, and pre‑ and post‑survey differences were analyzed using paired t‑tests with a two‑sided α = 0.05. Given the exploratory nature of the evaluation, no adjustments were made for multiple comparisons, and interpretation focused on the magnitude of effect sizes. Effect sizes were calculated using Cohen’s dᶻ for paired samples. Statistical analysis was conducted using Stata version 18 (StataCorp, College Station, TX). The study was reviewed by the University of Pittsburgh Institutional Review Board and determined to be exempt.

## Results

All interns who participated in the curriculum completed pre- and post-surveys (35/35, 100% response rate). Most interns were 25-29 years old (30/35, 86%), were planning on a subspecialty career (26/35, 74%), and had not previously received invisible illness communication strategy teaching (31/35, 89%). Interns felt communication was an important aspect of working with patients with invisible illness before and after the curriculum (4.86 vs. 4.91, p = 0.16) (Table 1). Following the workshop, interns reported a statistically significant reduction in perceived communication difficulty (4.09 vs. 3.51, p < 0.01) and a statistically significant increase in looking forward to working with patients with invisible illness (2.23 vs. 3.20, p < 0.01) (Table 1). There was a statistically significant improvement across all assessed comfort items (Table 1). Small-to-moderate effect sizes were observed for interns’ increased comfort with expressing empathy (4.37 vs. 4.63, p = 0.02, Cohen’s dᶻ = 0.42) and validation (4.00 vs. 4.43, p < 0.01, Cohen’s dᶻ = 0.70), whereas large effect sizes were observed for the other communication skills. The largest effects were seen for providing education (2.51 vs. 3.89, p < 0.01, Cohen’s dᶻ = 1.56), setting expectations (2.54 vs. 4.09, p < 0.01, Cohen’s dᶻ = 1.89), and discussing management strategies (2.51 vs. 3.8, p < 0.01, Cohen’s dᶻ = 1.50). The session was rated as excellent by 71% (25/35) and good by 29% (10/35) of interns.

**Table 1:** Analysis of internal medicine interns’ pre- and post-survey invisible illness communication strategy curriculum responses. NS = not significant.

Survey items	Pre-Mean (SD)	Post-Mean (SD)	Mean difference (95% CI)	p-value	Cohen’s dᶻ
Attitudes					
Communication is an important aspect of working with patients with invisible illnesses.	4.86 (0.36)	4.91 (0.28)	0.06 (0.02 to 0.14)	NS	0.24
I find communication with patients with invisible illnesses difficult.	4.09 (0.82)	3.51 (0.78)	-0.57 (-0.90 to -0.25)	<0.01	0.60
I look forward to working with patients with invisible illness.	2.23 (0.43)	3.20 (0.68)	0.97 (0.68 to 1.27)	<0.01	1.13
Comfort levels I feel comfortable…					
Performing an evaluation for a patient who is presenting with a possible invisible illness	2.83 (0.79)	3.97 (0.57)	1.14 (0.86 to 1.42)	<0.01	1.41
Expressing empathy to a patient with an invisible illness	4.37 (0.55)	4.63 (0.50)	0.26 (0.05 to 0.47)	0.02	0.42
Validating concerns of a patient with an invisible illness	4.00 (0.64)	4.43 (0.56)	0.43 (0.22 to 0.64)	<0.01	0.70
Communicating a diagnosis of invisible illness to a patient	2.74 (1.01)	4.00 (0.64)	1.26 (0.94 to 1.57)	<0.01	1.37
Educating a patient about invisible illnesses	2.51 (0.98)	3.89 (0.76)	1.37 (1.07 to 1.67)	<0.01	1.56
Setting expectations with a patient with invisible illnesses	2.54 (0.85)	4.09 (0.56)	1.54 (1.26 to 1.82)	<0.01	1.89
Discussing management strategies with a patient with invisible illnesses	2.51 (0.89)	3.8 (0.72)	1.28 (0.99 to 1.58)	<0.01	1.50
Responding to questions from a patient with invisible illnesses	2.51 (0.92)	3.71 (0.83)	1.2 (0.87 to 1.53)	<0.01	1.24

In the free-response section, interns noted the practical framework, real-world sample phrasing, case-based discussion, and normalization of the challenging nature of invisible illness as helpful. Areas for improvement included dedicated time for practice conversations, additional sessions on invisible illness diagnosis and management, and increased focus on the patient's perspective. We did not perform a formal qualitative analysis on free-response feedback; however, in Table 2, we provide examples of free-response feedback.

**Table 2:** Selected examples of internal medicine interns’ invisible illness curriculum post-survey free response answers.

Helpful aspects of the curriculum	Free response answers
Practical framework	“I liked the provided framework of symptoms > diagnosis > management with the ‘5 Es.’ I think it's general enough that I could see myself using it in many situations but specific enough that I have ideas for what to actually do in my own clinical practice.” – Intern 20
	“Gives a straightforward framework on how to address these types of cases.” – Intern 35
Real-world sample phrasing	“I liked the specific framework and examples of communication strategies that were provided. Everything felt actionable.” – Intern 2
	“Loved examples of how to say things.” – Intern 7
	“Great curriculum, gave me tools on how to communicate and set expectations.” – Intern 24
Case-based discussion	“I really liked how a case was used to illustrate the lecture.” – Intern 19
	“I liked using the case as a framework” – Intern 32
Normalizing the challenging nature of invisible illness	“I appreciated that Dr. [faculty member] acknowledged the challenges involved with managing invisible illness - [they] normalized that for us.” – Intern 2
Areas of improvement in the curriculum	
More time for practice conversations	“It may benefit from role play - practicing some of the communication strategies in real time….” – Intern 5
	“I think it could be helpful to run through drills for how to have difficult conversations about invisible illness with patients.” – Intern 3
	“More time for skills session to actually practice saying the words.” – Intern 18
Additional sessions on invisible illness diagnosis and management	“Include more history on symptoms burden. It felt like a leap to diagnosis…. I don't feel comfortable in diagnosing which is a big hurdle to the whole exercise, so reviewing the diagnostic criteria would be helpful.” – Intern 24
	“I think this session changed the way I feel about seeing such patients. If we can get more knowledge about such functional disorders (I feel like I don't know enough about… disorders) it will be great. – Intern 23
	“Talk about meds to offer.” - Intern 29
Increased focus on the patient perspective	“Could be helpful to hear some patient stories/perspectives.” -Intern 14

## Discussion

To our knowledge, this is the first study to evaluate an invisible illness communication strategy curriculum. The curriculum was well received by IM interns and increased comfort levels across all assessed communication skills. Small effect sizes were seen for items such as comfort with expressing empathy and validation, which may be a result of the ceiling effect, as participants expressed high levels of comfort with these items at baseline. Limitations of the study include the single-institutional design, small sample size without a control group, self-reported outcomes, use of a de novo survey with limited psychometric validation, lack of long-term follow-up, and lack of objective skills assessment. The single institutional design and small sample size may have negatively impacted the validity of the study findings and may limit generalizability to other institutions or geographic regions. Additionally, there may be limited generalizability to primary care-oriented programs, as 74% of the participants were planning a subspecialty career. The small group setting and dedicated time with one faculty member may have created social desirability bias, influencing participants to respond positively to the post-survey. We attempted to mitigate any bias by anonymous survey responses; however, social desirability bias may have substantially impacted responses.

Related studies in chronic, noncancer pain, medically unexplained symptoms, and fibromyalgia demonstrate that educational interventions are generally well-received by physicians and improve knowledge, skills, and attitudes [38-44], but these curricula are infrequently taught in medical education despite the prevalence of invisible illness. Poor communication in the care of patients with invisible illness has far-reaching consequences for both patient and provider outcomes [26-28]. The current study assesses participants’ attitudes and comfort with communication skills when working with patients with invisible illness; however, the study does not assess whether increased invisible illness communication strategy education leads to application of this knowledge, behavior change, or impact on patient outcomes. To address this important gap, the next steps would include designing a study to assess the educational impact on objective outcomes, such as the application of the framework during a visit or patient satisfaction with the provider’s communication. Although additional educational interventions, including longitudinal, consistent education across all levels of medical training, are likely needed to train physicians to be fully competent in invisible illness, the invisible illness communication strategy curriculum represents a feasible first step. We believe interns may be a particularly important group to target for invisible illness communication strategy education, as they have likely gained sufficient clinical exposure to recognize these challenges, but have not yet developed fixed, negative attitudes toward caring for patients with invisible illness.

## Conclusions

The current study demonstrates that a one-hour invisible illness communication strategy curriculum was feasible, acceptable to IM interns, and had perceived value among the participants; however, it was unable to assess participant behavior change or impact on patient outcomes. Future studies could further evaluate the impact of this curriculum, including trainees’ application of the framework during clinical encounters, trainees’ invisible illness attitudes over the course of residency, and whether implementation is associated with improved patient-physician relationships, patient and physician satisfaction, and patient-level outcomes. We plan to incorporate intern feedback in subsequent iterations of this workshop by featuring patient quotations from qualitative studies throughout the presentation to highlight the patients' perspectives and reserving time at the end of the curriculum to practice communication drills. Of note, several interns requested more invisible illness education in the free-response section. Exploring existing invisible illness curricula in medical education and considering additional development may be necessary to train physicians competent in caring for patients with invisible illness and improve healthcare experiences and outcomes for patients with invisible illness.

## Appendices

Appendix 1: Facilitator guide

Invisible Illness Communication Strategy: The 5 Es Framework

Introduction - 5 minutes

Review the following key points:

Definition of invisible illness: Clinical syndromes that are not readily visible or measurable by physical exam or diagnostic testing, and patients outwardly appear healthy.

Examples of invisible illnesses: Fibromyalgia, myalgic encephalomyelitis/chronic fatigue syndrome (ME/CFS), and disorders of gut-brain interaction (e.g., irritable bowel syndrome).

Impact of invisible illnesses: Impaired physical and mental health, decreased work and home productivity, low quality of life, costs to the individual and the healthcare system.

How patients with invisible illness experience the healthcare system: Stigma, provider lack of belief or mistrust of patient-reported symptoms, being told “nothing is wrong”/testing is normal without additional discussion or follow-up, healthcare odyssey to diagnosis (multiple specialist visits, long wait times, multiple visits without discussion of a definitive diagnosis).

Case presentation - 15 minutes

You are paged with a new admission from the ED…

Rachel is a 21-year-old female with a past medical history of major depressive disorder and two recent hospital admissions for nausea, vomiting, and abdominal pain who presents again with nausea, vomiting, and abdominal pain. Patient has previously been evaluated by gastroenterology and has had an extensive prior workup, including complete blood count (CBC), comprehensive metabolic panel (CMP), erythrocyte sedimentation rate (ESR), C-reactive protein (CRP), celiac panel, urine drug screening (UDS), CT of the abdomen and pelvis with contrast, esophagogastroduodenoscopy (EGD), gastric emptying study, non-contrasted head CT, and urine porphobilinogen, all of which have been unremarkable. At prior admissions, the patient improved with supportive care, including pain control, antiemetics, and IV fluids.

You go to see Rachel after she arrives at the ward from the ED. History is obtained from Rachel and her mother. Rachel reports that nothing helps her abdominal pain other than IV pain medication. Rachel’s mother reports that all of her testing has been normal, and the gastroenterologists cannot find anything to explain her symptoms. On review of gastroenterology notes, a diagnosis of cyclic vomiting syndrome vs. visceral hypersensitivity was considered. Rachel and her mother report they have never heard these terms and have been told, “nothing is wrong.” They are very frustrated. Rachel feels like the medical staff have suggested she is a drug seeker, and it is “all in her head.”

Ask the learners to consider and respond to the questions below in the context of this case. Write the main points from learner responses on the board (if available).

What is your emotional reaction to this case?

Do you feel like you are equipped to manage this case? Why or why not?

Are you looking forward to seeing and working with this patient? Why or why not?

What do you anticipate being challenging?

Importance of communication - 5 minutes

Review the following key points:

Pain invalidation has been linked to increased pain intensity, threats to moral integrity, impaired psychological and physical functioning, and emotional distress.

Empathetic, patient-centered communication has been linked to increased patient satisfaction, empowerment, resilience, and self-efficacy, strong patient-physician relationships, better adherence to treatment plans, and improved patient outcomes.

Presentation of the 5 Es framework and key concepts by skill - 25 minutes

Review the 5 Es framework along with key concepts in each skill, and pair this with example phrasing. Review how the skills pair with the patient’s journey, starting with the symptoms/diagnostic phase to diagnosis, and then to management and follow-up.

Evaluate (patient’s journey: symptoms/diagnostics phase)

- Use open-ended questions

- Active listening

- Display genuine curiosity by asking follow-up/clarifying questions

- Explore prior interaction with the healthcare system

- Perform a holistic evaluation by asking about symptom impact on sleep, mood, personal and professional life, and goals

- Review prior work-up and critically evaluate whether additional work-up is appropriate

- Review prior treatment modalities and patient response/outcome

Empathize and validate (patient’s journey: symptoms/diagnostics phase)

- Use empathetic and validation statements

- Address prior invalidating experiences

- Dispel myths or prior inaccurate medical advice

Educate (patient’s journey: diagnosis)

- Address prior test results

- Discuss diagnostic criteria for the condition under consideration

- Give the patient as specific a diagnosis as possible

- Assess the patient’s response to diagnosis

- Discuss what is known and not known about the diagnosis

- Use lay language

Set Expectations (patient’s journey: diagnosis)

- Discuss the natural history of the condition

- Discuss treatment options and the expected efficacy of treatment options

- Discuss and set realistic goals

Engage in care (patient’s journey: management and follow-up)

- Treat the patient as a collaborator

- Engage in an open discussion about treatment options

- Invite the patient to share their opinions and ask questions

Return to the case - 5 minutes

Following the presentation of the 5 Es framework, revisit the case and discuss where the patient is on her journey and what skills would be most appropriate to apply at this time point. Have the learners practice what words they would actually use when speaking to Rachel and her mother. Revisit and address any points previously written on the board that were not addressed during the 5 Es presentation framework.

Wrap-up - 5 minutes

Invite participants to ask any questions or bring up other points for discussion. 

If time allows can review additional challenging statements or provide this as a take-home handout for participants.

Additional challenging patient statements and sample responses

Patient statement: “Something else is going on. I don’t believe in [diagnosis].”

Sample responses:

Patient has previously had an extensive evaluation:

· “We still have a lot of gaps in our knowledge and understanding of [diagnosis]. For now, I/we do believe in [diagnosis] and what you’re telling me seems to fit with it.”

· “I would be happy to work with you on some [diagnosis] specific therapies while you explore a second opinion at the same time.”

· “It can be hard to receive a diagnosis you feel unsure about. I would also be happy to provide you with some more information to take home and look over and we can continue this discussion at a follow up appointment as well.”

· “It sounds like this wasn’t what you were expecting. What do you feel I am missing?”

Patient has not previously had an extensive and/or appropriate evaluation:

· “A lot of what you are telling me does fit with [diagnosis], but I agree that something else may be going on. I would like to check some additional lab work to rule out some other possibilities.”

Patient statement: “The only thing that works for me is oxycodone. I am not interested in other medications.”

Sample responses:

· “I hear you and I believe you that oxycodone helps with your pain. I want to treat your pain; however, as a provider I must consider the downsides as well.”

· “I appreciate you telling me what has worked for you in the past. Right now, I think the risks of oxycodone ultimately outweigh the benefits. I am happy to discuss other options with you and work together over time to improve your pain.”

· “If you are willing to consider another medication, we can do close follow-up visits and work together to find the best solution for you. I am also available via phone or messenger in between visits if you have concerns.”

Appendix 2: Invisible illness communication strategy curriculum assessment

Pre-curriculum Survey

Thank you for agreeing to participate!

What is an invisible illness? We define an invisible illness as a health condition(s) that is not immediately visible to others, including healthcare professionals, is a clinical diagnosis, and is not associated with abnormal diagnostic testing (labs, imaging, other procedures, etc.). Examples of invisible illnesses include fibromyalgia, myalgic encephalomyelitis/chronic fatigue syndrome (ME/CFS), irritable bowel syndrome, migraines, etc.

Pre-curriculum survey

Please indicate your level of agreement with the following statements.

**Table 3:** Pre-curriculum survey.

Statement	Strongly disagree, 1	Disagree, 2	Neutral, 3	Agree, 4	Strongly agree, 5
General perceptions					
Communication is an important aspect of working with patients with invisible illnesses.	1	2	3	4	5
I find communication with patients with invisible illnesses difficult.	1	2	3	4	5
I look forward to working with patients with invisible illness.	1	2	3	4	5
Comfort levels - I am comfortable:					
Performing an evaluation for a patient who is presenting with a possible invisible illness	1	2	3	4	5
Expressing empathy to a patient with an invisible illness	1	2	3	4	5
Validating concerns of a patient with an invisible illness	1	2	3	4	5
Communicating a diagnosis of invisible illness to a patient	1	2	3	4	5
Educating a patient about an invisible illness	1	2	3	4	5
Setting expectations with a patient with an invisible illness	1	2	3	4	5
Discussing management strategies with a patient with an invisible illness	1	2	3	4	5
Responding to questions from a patient with an invisible illness	1	2	3	4	5

1. Have you previously received teaching about communication skills for an invisible illness?

• Yes • No

2. What is your current age?

• 20-24 • 25-29 • 30-34 • 35-39 • 40+

3. What are your current career plans after residency?

• Subspecialty Fellowship

• Academic Hospitalist

• Academic Primary Care

• Community Hospitalist

• Community Primary Care

• Other: __________________________________

STOP HERE UNTIL THE END OF THE WORKSHOP

Invisible Illness Communication Strategy Curriculum Assessment

Post-curriculum survey

Please indicate your level of agreement with the following statements:

**Table 4:** Post-curriculum survey.

Statement	Strongly disagree, 1	Disagree, 2	Neutral, 3	Agree, 4	Strongly agree, 5
Attitudes					
Communication is an important aspect of working with patients with invisible illnesses	1	2	3	4	5
I find communication with patients with invisible illnesses difficult	1	2	3	4	5
I look forward to working with patients with invisible illnesses	1	2	3	4	5
Comfort levels - I am comfortable:					
Performing an evaluation for a patient who is presenting with a possible invisible illness	1	2	3	4	5
Expressing empathy to a patient with an invisible illness	1	2	3	4	5
Validating concerns of a patient with an invisible illness	1	2	3	4	5
Communicating a diagnosis of invisible illness to a patient	1	2	3	4	5
Educating a patient about invisible illnesses	1	2	3	4	5
Discussing expectations with a patient with an invisible illness	1	2	3	4	5
Discussing management strategies with a patient with an invisible illness	1	2	3	4	5
Responding to questions from a patient with an invisible illness	1	2	3	4	5

I would rate the overall quality of this session as:

• Excellent • Good • Average • Poor • Very Poor

What was helpful about this session?

What should be changed for future sessions?
